# Hybrid Metal Graphene-Based Tunable Plasmon-Induced Transparency in Terahertz Metasurface

**DOI:** 10.3390/nano9030385

**Published:** 2019-03-06

**Authors:** Xianjun Wang, Hongyun Meng, Shuying Deng, Chaode Lao, Zhongchao Wei, Faqiang Wang, Chunhua Tan, Xuguang Huang

**Affiliations:** Guangdong Provincial Key Laboratory of Nanophotonic Functional Materials and Devices, School of Information and Optoelectronic Science and Engineering, South China Normal University, Guangzhou 510006, China; xianjunwang@m.scnu.edu.cn (X.W.); shuying@m.scnu.edu.cn (S.D.); laochaode@m.scnu.edu.cn (C.L.); wzc@scnu.edu.cn (Z.W.); fqwang@scnu.edu.cn (F.W.); tch@scnu.edu.cn (C.T.); huangxg@scnu.edu.cn (X.H.)

**Keywords:** graphene metamaterials, plasmon-induced transparency, slow light

## Abstract

In this paper, we look at the work of a classical plasmon-induced transparency (PIT) based on metasurface, including a periodic lattice with a cut wire (CW) and a pair of symmetry split ring resonators (SSR). Destructive interference of the ‘bright-dark’ mode originated from the CW and a pair of SSRs and resulted in a pronounced transparency peak at 1.148 THz, with 85% spectral contrast ratio. In the simulation, the effects of the relative distance between the CW and the SSR pair resonator, as well as the vertical distance of the split gap, on the coupling strength of the PIT effect, have been investigated. Furthermore, we introduce a continuous graphene strip monolayer into the metamaterial and by manipulating the Fermi level of the graphene we see a complete modulation of the amplitude and line shape of the PIT transparency peak. The near-field couplings in the relative mode resonators are quantitatively understood by coupled harmonic oscillator model, which indicates that the modulation of the PIT effect result from the variation of the damping rate in the dark mode. The transmitted electric field distributions with polarization vector clearly confirmed this conclusion. Finally, a group delay $t_{g}$ of 5.4 ps within the transparency window is achieved. We believe that this design has practical applications in terahertz (THz) functional devices and slow light devices.

## 1. Introduction

Plasmon-induced transparency (PIT) is achieved with the same properties of the traditional EIT that allow a distinct transparency window in broad absorption or transmission spectrum [1], also known as the analogue of electromagnetically induced transparency (EIT-like) [2,3,4,5]. The strong dispersion at the transparent window can significantly slow down photons [6,7]. The PIT effect can be mimicked by non-quantum design approaches, such as plasmonic metamaterials [8,9,10,11,12], waveguides [13], photonic crystals [14], and gratings [15,16], which avert the stringent experimental requirements in implementations of practical applications.

In general, PIT behavior mainly from two different destructive interference mechanisms, including the result of engaging “trapped mode” resonances [4,17] or by the eigenmodes resonances in the near field coupling of ‘bright’ and ‘dark’ [3,18]. In developing a strong PIT phenomenon, the close resonance frequencies and distinct quality (Q) factors of the relative modes are necessary. The ‘bright’ mode resonator generally is excited by strong coupling with the radiation field (low Q factor) whereas weakly coupling in the ‘dark’ mode (large Q factor). However, the ‘dark’ mode can be excited by ‘bright’ mode with the near-field coupling when close proximity resonance frequency in the relative modes, which results in an extremely narrow window in reflection or transmission spectrum that we call EIT-like [17,19].

In practice, an active modulation of the PIT response, to achieve the well-controlled group delay, as well as many other practical function units, such as plasmonic switching, are highly attractive. Gu et al. achieved the complete modulation of EIT effect in classical metamaterials by photoactive Si-integrated functional unit cells and the optical pump-terahertz probe (OPTP) measurement methods [8]. Xu et al. presented the same control with a different structure [20]. Wu et al. realized an optically precise and active modulation of terahertz wave by adjusting the optical pump pulse on EIT metamaterials [21]. In practice, however, the limited response time of the excited carriers in Si islands (~1 ms), or the difficulty in precisely controlling the refractive index of dielectric substrates, severely hinders the implementations of ultrafast optoelectronics applications. Graphene, a two-dimensional (2D) material, having a lower carrier relaxation time [22,23,24] and a negligible insertion loss [25,26] is promising for an ultrafast response. The tunability of its surface conductivity, achieved by changing the Fermi energy level with chemical doping or electrostatic gating [27,28], has taken a wide interest in active modulation of the PIT response [29,30,31,32,33,34,35,36,37,38]. For example, Zhao et al. proposed a graphene-based PIT metasurface and achieved tunability by changing the Fermi level of the integrated graphene strip [29]. Nevertheless, the isolated graphene strip in per unit cell is hard to be precise in control and in practice. Xiao et al. integrated a continuous graphene strip into a metal-based terahertz metasurface and actively tuned the damping rate of the ‘dark’ mode resonators to achieve active modulation of the EIT-like response [39]. This method perfectly realizes expedient tuning of the graphene resonator, but the complicated structure is still an issue.

In this paper, we propose a design of a classical Al-based terahertz metasurface, consisting of a CW element and an SSR element on a semi-infinite Si-on-sapphire substrate, to achieve PIT effects. The CW serves as an electric dipole antenna to support the ‘bright’ mode resonance, whereas the SSR serves to support the ‘dark’ mode by being indirectly excited with a far-field coupling. We further discussed the resonance mechanism of the destructive interference in ‘bright-dark’ modes with respect to the electric field and surface current distributions. With simulations, we explore the effects of the coupling distance between the relative modes resonators, and the vertical distance of the split gap on the PIT effect. Furthermore, we integrate a continuous graphene strip monolayer into the metamaterial, and realize an active control of the amplitude and line shape of the PIT transparency peak, by manipulating the Fermi level of graphene. The coupled harmonic oscillator model and nonlinear fitting are applied to quantitatively understand the near-field coupling in the relative modes. It is shown that the modulation of the PIT effect results mainly from changes in the damping rate of the dark mode, and the transmitted electric field distributions with the polarization vector clearly confirms this conclusion. Finally, a group delay $t_{g}$ of 5.4 ps within the transparency window, which corresponds to 1.62-mm distance of free space propagation is achieved. We believe that the design has practical applications in slow light devices and terahertz functional devices.

## 2. Structural Design and Numerical Mode

**Figure 1:**
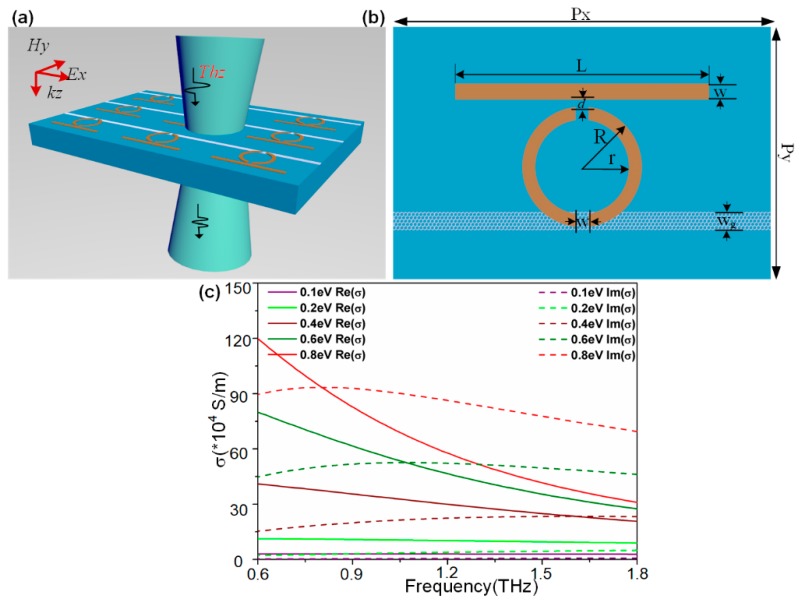
(**a**) Schematic of the proposed PIT metasurface with polarization configuration of incident light. (**b**) Geometrical description of a unit cell: *Px* = 120 μm, *Py* = 80 μm, *L* = 80 μm, *W* = 5 μm, *r*_1_ = 23 μm, *r*_2_ = 18 μm, *d* = 3 μm, *W_g_* = 7 μm, and △*h* = 0 μm (△*h*: vertical distance of split gap, not shown in figure). (**c**) Real and imaginary parts of the graphene conductivity with the Fermi level increasing from 0.1 eV to 0.8 eV.

The metamaterial, with classical structure (similar to [19,40]) but different split-direction of the ring resonator, for the realization of EIT with ‘bright-dark’ resonant mode is schematically illustrated in Figure 1a. The unit cell of the structure, shown in Figure 1b, consists of a cut wire (CW) resonator and a pair of symmetric split ring resonators (SSR), with a continuous graphene ribbon monolayer deposited between the SRR-pair and the Si-on-sapphire substrate. Aluminum is chosen as the resonators material, which can be characterized by the Drude model in the THz regime [41]:

(1) $$\varepsilon_{Al}=\varepsilon_{\infty }-\frac{\omega_{p}^{2}}{\omega^{2}+i\omega \gamma }$$

Here the parameters $\omega_{p}=2.24\times 10^{16}\;\mathrm{rad}/s$ and $\gamma =1.22\times 10^{14}\;\mathrm{rad}/s$ represent the plasma frequency and the damping constant, respectively. The semi-infinite Si-on-sapphire substrate consists of a 0.5-µm-thick Si film (n=3.42) and 499.5-µm-thick sapphire islands (n=1.78). 

The conductivity of the graphene monolayer *σ_g_* is derived from the intra-band and inter-band transitions, which can be expressed as $\sigma_{g}\left(\omega \right)=\sigma_{intra}\left(\omega \right)+\sigma_{inter}\left(\omega \right)$ [42]. According to the Pauli exclusion principle [43], the contribution to the conductivity *σ_g_*, mainly comes from the intraband process in the lower THz band [44]:

(2) $$\sigma_{g}\left(\omega \right)=\sigma_{intra}\left(\omega \right)=i\frac{e^{2}k_{B}T}{\pi \hbar^{2}\left(\omega +i\tau^{-1}\right)}\left(\frac{E_{F}}{k_{B}T}+2\ln\left(e^{\frac{-E_{F}}{k_{B}T}}+1\right)\right)$$

Here *ℏ* and *e* are Planck constant and the electron charge, respectively. τ represents carrier relaxation time expressed with $\tau =\mu E_{F}/ev_{F}^{2}$, which is related to electron charge carrier mobility μ, the Fermi level $E_{F}$ and the Fermi velocity $v_{F}$. $\sigma_{g}$ can be further simplified assuming high doping in graphene, with the Fermi level $E_{F}\gg k_{B}T$ and $E_{F}\gg \hbar \omega$, which can be expressed by the Drude-like model [45,46]:

(3) $$\sigma_{g}\left(\omega \right)=\frac{e^{2}E_{F}}{\pi \hbar^{2}}\frac{i}{\omega +i\tau^{-1}}$$

In calculations, we set $\mu =3000\;{\mathrm{cm}}^{2}/V\cdot s$ and $v_{F}=1.1\times 10^{6}\;m/s$, which is consistent with Reference [39]. The real and imaginary parts of the graphene conductivity obtained from the simulation with the Fermi level increasing from 0.1 eV to 0.8 eV is shown in Figure 1c.

The 3D finite-difference time-domain (3D-FDTD) method was employed in our work. Periodic boundary conditions are applied in the *x* and *y* directions, and perfectly matching layers are applied in the *z* direction. In calculations, good convergence of the calculated result can be obtained with the non-uniform mesh setting.

## 3. Results and Discussions

**Figure 2:**
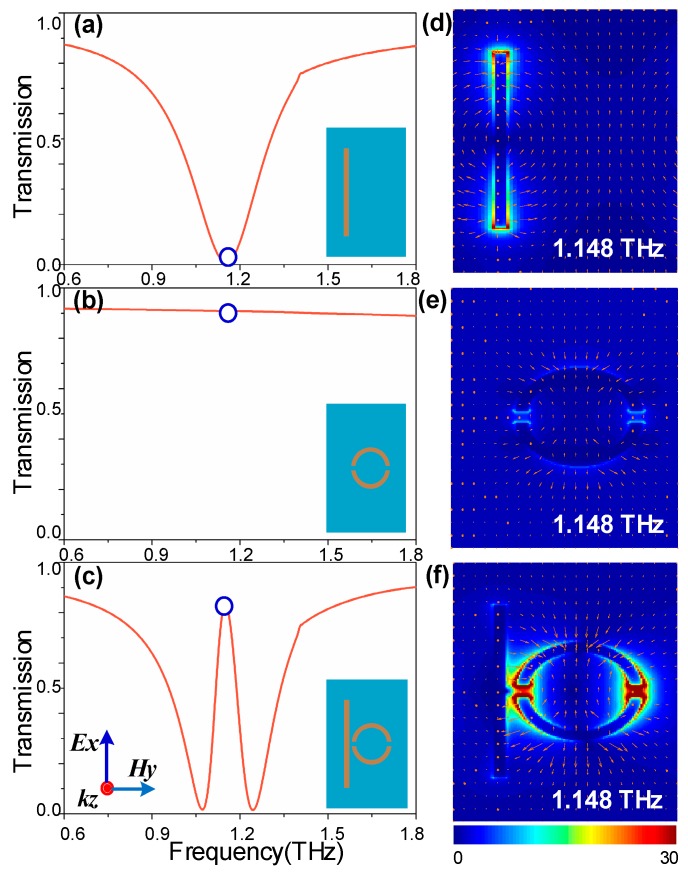
(**a**–**c**) Measured transmission profile of the independent CW, SSR-pair, and the PIT metasurface; the insets show the structural samples with the polarization illustration. (**d**–**f**) Transmitted electric field distributions probed in the corresponding structure at resonant frequency of 1.148 THz; the polarization vector is indicated by arrows.

To investigate the resonant features of the Al-based metamaterials without graphene integration, the corresponding transmitted profile of the individual CW arrays, SSR-pair arrays, and the combination PIT metasurface, are shown in Figure 2a–c, respectively. A localized surface plasmon (LSP) resonance mode is observed at 1.148 THz, with a 3 dB bandwidth of 0.35 THz in the CW array, which is caused by the radiation coupling to the incident E-field oriented forward the CW direction (a TE wave). On the other hand, the SSR-pair serves as subradiant mode at the corresponding frequency due to the weak coupling in the condition of structural symmetry with respect to the polarization of the incident plane wave. When the CWs and the SSRs are integrated in a unit cell, an EIT-like transparency window with over 85% transmission is achieved within the original broad stop band (bright mode) located at 1.148 THz.

**Figure 3:**
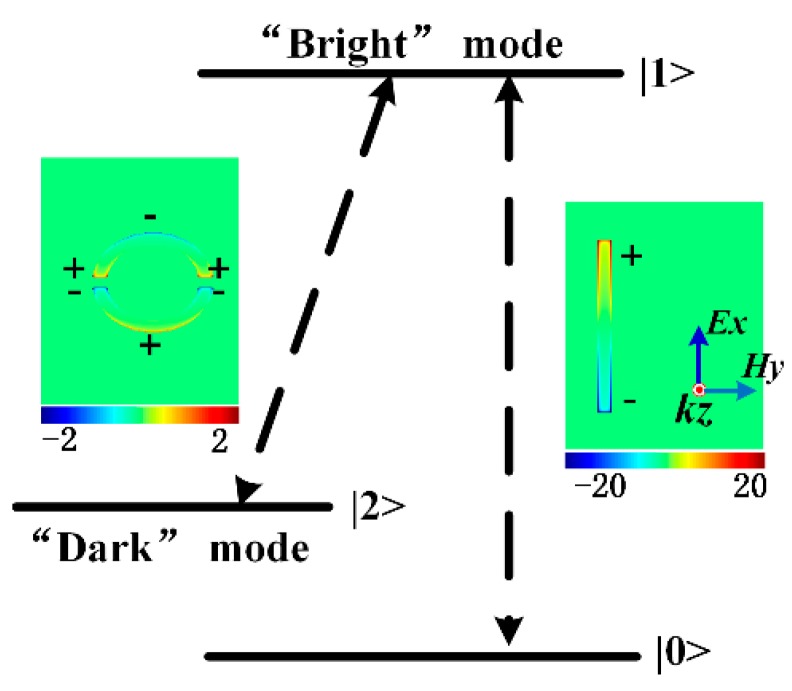
Schematic of interference with the classical 3-level resonant system. Inset: z-component of electric field (*E_z_*) distributions of the ‘dark’ (**left**) and ‘bright’ (**right**) modes.

To further explain the physical mechanism underlying the PIT effect, the transmitted electric field distributions of the cross section at 1.148 THz resonant frequency with polarization vectors are shown in Figure 2d–f. The classical 3-level resonant system [47] and the *z*-component of the electric field (*E_z_*) distributions (insets in Figure 3) were applied to illustrate the interference between the radiative and subradiant modes. As illustrated in Figure 3, the CW serves as an electric dipole antenna to support the ‘bright’ mode resonance. Due to the collective oscillations of the radiative resonator, a very strong enhancement of the electric field, concentrating on the edges and corners of the CW, is observed, which is shown in Figure 2d. The indirectly excited subradiant mode with a weak far-field coupling is shown in Figure 2e. However, it can interact with the ‘bright’ mode through near-field coupling [3,19], resulting in a distinct transparent window with destructive interference originating from the π phase difference in the relative modes [3,48]. It is clear that the fields in the radiative resonator are suppressed, whereas subradiant resonators are excited by near-field coupling with a distinct enhancement of the electric field, shown in Figure 2f. From a quantum point of view [49,50], it is produced when the two pathways |0〉-|1〉 and |0〉-|1〉-|2〉-|1〉 destructively interfere, as shown in Figure 3. The |0〉-|1〉 corresponds to the directly excited process of the ‘bright’ mode, whereas |0〉-|2〉 corresponds to a forbidden transition of the ‘dark’ mode. However, near-field coupling between the relative modes offers the possibility to achieve excitation state |2〉 of the ‘dark’ mode.

**Figure 4:**
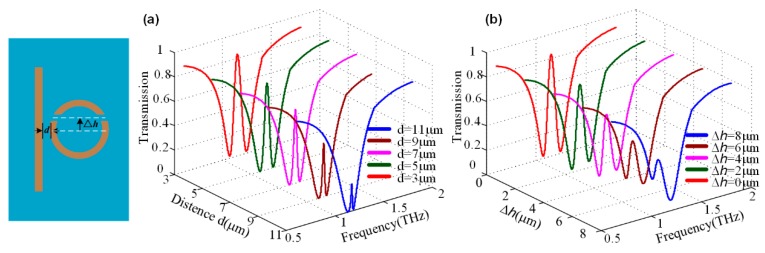
Transmittance spectra of arrays (**a**) with different separation distance between ‘bright’ and ‘dark’ resonators and (**b**) with different vertical distance of the split gap △*h.* A schematic diagram of the sample is shown on the left.

As illustrated above in Figure 3, ‘dark’ modes are excited by near-field coupling with the ‘bright’ mode. Therefore, we further explore the coupling strength affecting on the PIT effect. There are two ways to change the coupling coefficient: varying *d* or △*h*. Figure 4a illustrates the transmission spectrum with different separation distances *d* between the radiative and subradiant resonators. The schematic diagram of the sample is shown on the left. At *d* = 11 μm, the contrast of the dip is very small due to the weak coupling in the relative modes. As *d* decreases from 11 μm to 3 μm (△*h* is kept at 0 μm), the dip widens and becomes deeper, which confirms that the spatial separation determines the strength of near-field coupling [3]. Similar modulation of EIT amplitudes is also observed as △*h* varies from 0 μm to 8 μm (*d* is kept at 3 μm). The transmission dip on the resonance frequency is clearly lowered as the split gap deviates from its own center. As △*h* increases, the overall coupling coefficient decreases with the breaking of the symmetry of the dark atom structure.

**Figure 5:**
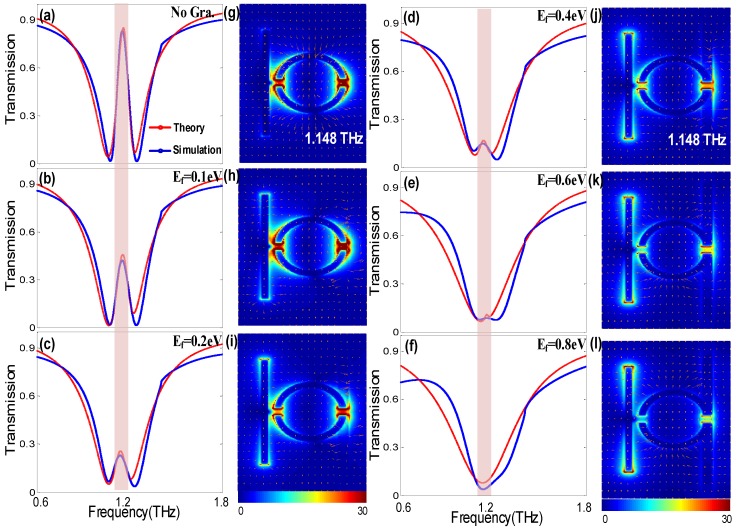
(**a**–**f**) Transmission profile of the hybrid metamaterials and the corresponding analytical fitted curves with increasing Fermi level of graphene. (**g**–**l**) Corresponding transmitted electric field distributions at 1.14 THz resonant frequency; the polarization vector is indicated by arrows.

Apart from amplitude modulation of EIT, based on adjusting the coupling coefficient by structural reset method, a continuous graphene monolayer is integrated in the original structure to achieve dynamic modulation of the EIT effect. Here, a 7 μm wide graphene strip is deposited between the originally subradiant resonator (SSR) and the Si-on-sapphire substrate, as shown in Figure 1b. With this configuration, it is easy to fabricate and realize electric tuning of the graphene wires by controlling the bias voltage between the substrate and the electrode. Figure 5b–f show the transmission spectra of the integrated metasurface with different Fermi level *E_f_* of the graphene wires (blue line). It is clearly seen that a complete on-to-off modulation of the PIT transparent window can be achieved at the original resonant frequency. Compared to the configuration without graphene, in Figure 5a the transmission amplitude of the PIT peak has an obvious decrease with approximately 50% transmission when the Fermi level is *E_f_* = 0.1 eV. With the Fermi level increasing to 0.8 eV, the PIT transparency peak gradually disappears, leaving an LSP-like resonance dip in the transmission spectrum. It is worth noting that the modulation of the graphene Fermi level does not cause a notable frequency shift.

To further explain the underlying physical mechanism of graphene modulation on the effect of PIT, the corresponding transmitted electric field |E| distributions at 1.148 THz resonant frequency are shown in Figure 5g–l. As graphene Fermi level increases gradually, a strong enhancement of electric field from the SSRs resonator, transferring to CW resonator, which reflects a strong suppression of the ‘dark’ mode and an enhancement in the ‘bright’ mode. To further illustrate the nexus between the electric field redistribution and *E_f_* of graphene, the coupled harmonic oscillator model and nonlinear fitting are applied to quantitatively understand the near-field coupling in the relative modes. The destructive interference in the proposed PIT metasurface can be described by the following equations [3,4],

(4) $$\ddot{x_{1}}+\gamma_{1}{\dot{x}}_{1}+\omega_{0}^{2}x_{1}+\kappa x_{2}=gE_{0}e^{j\omega t}$$

(5) $$\ddot{x_{2}}+\gamma_{2}{\dot{x}}_{2}+{\left(\omega_{0}+\delta \right)}^{2}x_{2}+\kappa x_{1}=0$$

where the $x_{1}$ and $x_{2}$ are the resonance amplitudes of the ‘bright’ and ‘dark’ modes, respectively. $\omega_{0}=2\pi \times 1.148$ THz, and $\left(\omega_{0}+\delta \right)$ represent the central resonance frequency of the relative modes, where δ is the detuning frequency of the CWs and SSRs oscillators. $\gamma_{1}$ and $\gamma_{2}$ denote the damping rates, expressed by $\gamma =\gamma_{R}+\gamma_{NR}$, here $\gamma_{R}$, $\gamma_{NR}$ refer to radiative and non-radiative decay rates, respectively. The parameter *g* means the coupling strength of the ‘bright’ modes resonator with the incident light and κ is an effective coupling coefficient between the relative modes. 

According to Equations (4) and (5), the susceptibility χ of the EIT metamaterials can be calculated as [31,51]

(6) $$\chi =\chi_{r}+i\chi_{i}\propto \frac{\left(\omega -\omega_{0}-\delta \right)+i\frac{\gamma_{2}}{2}}{\left(\omega -\omega_{0}+i\frac{\gamma_{1}}{2}\right)\left(\omega -\omega_{0}-\delta +i\frac{\gamma_{2}}{2}\right)-\frac{\gamma^{2}}{4}}$$

where $\chi_{r}$ and $\chi_{i}$ refer to the dispersion and absorption within the metamaterial, respectively. Thus, the transmission *T* can be calculated by

(7) $$T=1-g\chi_{i}$$

**Figure 6:**
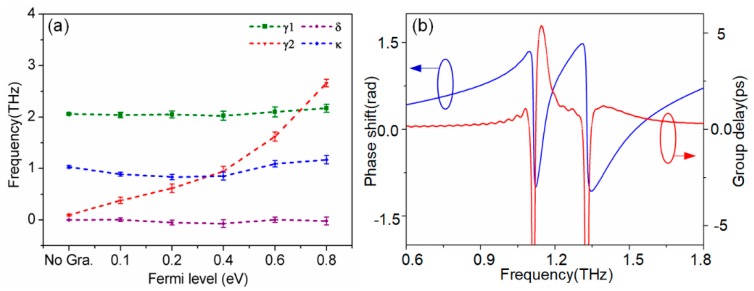
(**a**) Fitting parameters γ1, γ2, δ, and κ as a function of the Fermi level of graphene. (**b**) Transmission phase shift and group delay of the Al-based metamaterials without graphene.

The transmission spectra analytical fitted according to Equation (7) are shown in Figure 5, with red-dashed curves for direct comparison. It is evident that the simulation curves are in good agreement with the theoretical model. Table 1 displays the corresponding fitting parameters within the theoretical model. Furthermore, the fitting values for γ1, γ2, δ, and κ as a function of the graphene’s Fermi level are plotted in Figure 6a. The parameters δ, γ1, and κ are roughly constant, whereas the damping rate of ‘dark’ mode γ2 increases notably with increasing *E_f_*. Thus, the theoretical model indicates that the dynamical tunability of the PIT effect results from the change in the damping rate of the ‘dark’ mode. With increasing Fermi level, the surface conductivity of graphene σ increases, as shown in Figure 1c, resulting in an increase in the damping rate of the ‘dark’ mode resonator, composed by graphene strips and SSRs resonator. This is precisely due to the increase of damping rate weakening the strength of the near-field coupling between the relative modes, which results in a modulation of the PIT effect.

Slow light is one of promising findings in the generation of EIT phenomenon, resulting from strong dispersion at the transparent window [6,7]. Slow light capability is always described by the group delay $t_{g}$, which can be given by [13]

(8) $$t_{g}=\frac{d\varphi }{d\omega }$$

where φ is the transmission phase shift from the light source to the detected monitor. By calculating with the S parameters, the transmission phase shift of PIT metamaterial without the monolayer graphene is shown in Figure 6b (blue line). Furthermore, group delay $t_{g}$ calculated with Equation (8) is shown with the red-dash line. It is clearly seen that a group delay of 5.4 ps within the transparency window, which corresponds to 1.62-mm distance of free space propagation, can be achieved. This shows that the performance of the metamaterial on slow light device is achieved in this paper. Further performance comparisons to the current state of the art are shown in Table 2.

## 4. Conclusions

We presented a design of a classical Al-based metasurface consisting of a cut wire (CW) and a symmetric split ring resonator (SSR). The destructive interference of the ‘bright-dark’ mode originated from direct-excited plasmon resonance in the CW and the coupling excited resonance in the SSRs pair. In the simulation, we demonstrated that the relative distance *d* between the two resonators (CW and SSR) and the vertical distance *h* of split gap, play an important role in the coupling strength on the EIT effect. Furthermore, a complete modulation of the PIT system was shown by introducing a continuous graphene monolayer strip into the metamaterial. The coupled harmonic oscillator model and nonlinear fitting are applied to fully understand the near-field coupling in the relative modes. The theoretical analysis indicated that the dynamical tunable of the PIT effect arises from the change in the damping rate of the ‘dark’ mode. The vector diagram of the electric field clearly confirmed this conclusion. Finally, the largest group delay $t_{g}$ of 5.4 ps within the transparency window, which corresponds to 1.62-mm distance of free space propagation, is achieved. We believe that our design has practical applications in slow light devices and terahertz functional devices.

## Figures and Tables

**Table 1:** The corresponding fitting parameters γ1, γ2, δ and κ with different fermi level of the graphene.

*E_f_*	γ_1_	γ_2_	δ	κ
No Gra.	2.06	0.09398	0	1.031
0.1 ev	2.038	0.38	0.003	0.8877
0.2 ev	2.049	0.613	−0.05118	0.8346
0.4 ev	2.025	0.9447	−0.07163	0.85
0.6 ev	2.10	1.62	0.003	1.091
0.8 ev	2.17	2.66	−0.02	1.17

**Table 2:** The performance comparison of various PIT metamaterial structures with working band, Q-factor, modulation depth (MD), group delay.

Structure	Working Band	Q-Factor	Modulation Depth (MD)	Group Delay	Ref and Year
a CW, a pair of SRRs integrating photoactive silicon (Si)	0.4~1.2 THz		0.9 to 0.3		[8] 2012
a rectangular graphene antenna and a continuous graphene wire	2~5.0 THz	14.8	0.8 to 0.4		[29] 2016
a CW, asymmetric split ring (ASR) and a continuous graphene wire	0.7~1.2 THz		0.8 to 0.3	43 ps	[40] 2017
a CW, four U-shaped (USRs) and monolayer graphene sheets	0.5~1.5 THz		0.72 to 0	0.76 ps	[36] 2018
a CW, a pair of SRRs and a continuous graphene wire	0.4~1.0 THz		0.9 to 0.0	5.72 ps	[39] 2018
a CW, a pair of SRRs with graphene strips	0.2~1.0 THz		0.9 to 0.0	4.2 ps	[35] 2019
a CW, a SR resonator and a continuous graphene wire	0.8~2.3 THz		0.8 to 0.2		[38] 2019

## References

[1] Alzetta G. (1997). Induced transparency. Phys. Today.

[2] Papasimakis N., Fedotov V.A., Zheludev N., Prosvirnin S. (2008). Metamaterial analog of electromagnetically induced transparency. Phys. Rev. Lett..

[3] Zhang S., Genov D.A., Wang Y., Liu M., Zhang X. (2008). Plasmon-induced transparency in metamaterials. Phys. Rev. Lett..

[4] Liu N., Langguth L., Weiss T., Kästel J., Fleischhauer M., Pfau T., Giessen H. (2009). Plasmonic analogue of electromagnetically induced transparency at the drude damping limit. Nat. Mater..

[5] Liu N., Weiss T., Mesch M., Langguth L., Eigenthaler U., Hirscher M., Sonnichsen C., Giessen H. (2009). Planar metamaterial analogue of electromagnetically induced transparency for plasmonic sensing. Nano Lett..

[6] Lukin M., Imamoğlu A. (2001). Controlling photons using electromagnetically induced transparency. Nature.

[7] Longdell J.J., Fraval E., Sellars M.J., Manson N.B. (2005). Stopped light with storage times greater than one second using electromagnetically induced transparency in a solid. Phys. Rev. Lett..

[8] Gu J., Singh R., Liu X., Zhang X., Ma Y., Zhang S., Maier S.A., Tian Z., Azad A.K., Chen H.-T. (2012). Active control of electromagnetically induced transparency analogue in terahertz metamaterials. Nat. Commun..

[9] Devi K.M., Sarma A.K., Chowdhury D.R., Kumar G. (2017). Plasmon induced transparency effect through alternately coupled resonators in terahertz metamaterial. Opt. Express.

[10] Yahiaoui R., Manjappa M., Srivastava Y.K., Singh R. (2017). Active control and switching of broadband electromagnetically induced transparency in symmetric metadevices. Appl. Phys. Lett..

[11] Yahiaoui R., Burrow J.A., Mekonen S.M., Sarangan A., Mathews J., Agha I., Searles T.A. (2018). Electromagnetically induced transparency control in terahertz metasurfaces based on bright-bright mode coupling. Phys. Rev. B.

[12] Burrow J.A., Yahiaoui R., Sarangan A., Agha I., Mathews J., Searles T.A. (2017). Polarization-dependent electromagnetic responses of ultrathin and highly flexible asymmetric terahertz metasurfaces. Opt. Express.

[13] Lu H., Liu X., Mao D. (2012). Plasmonic analog of electromagnetically induced transparency in multi-nanoresonator-coupled waveguide systems. Phys. Rev. A.

[14] Yang X., Yu M., Kwong D.-L., Wong C.W. (2009). All-optical analog to electromagnetically induced transparency in multiple coupled photonic crystal cavities. Phys. Rev. Lett..

[15] Wei B., Liu H., Ren G., Yang Y., Ye S., Pei L., Jian S. (2017). Graphene based silicon–air grating structure to realize electromagnetically-induced-transparency and slow light effect. Phys. Lett. A.

[16] Lu H., Gan X., Mao D., Jia B., Zhao J. (2018). Flexibly tunable high-quality-factor induced transparency in plasmonic systems. Sci. Rep..

[17] Fedotov V., Rose M., Prosvirnin S., Papasimakis N., Zheludev N. (2007). Sharp trapped-mode resonances in planar metamaterials with a broken structural symmetry. Phys. Rev. Lett..

[18] Chiam S.-Y., Singh R., Rockstuhl C., Lederer F., Zhang W., Bettiol A.A. (2009). Analogue of electromagnetically induced transparency in a terahertz metamaterial. Phys. Rev. B.

[19] Yang Y., Kravchenko I.I., Briggs D.P., Valentine J. (2014). All-dielectric metasurface analogue of electromagnetically induced transparency. Nat. Commun..

[20] Xu Q., Su X., Ouyang C., Xu N., Cao W., Zhang Y., Li Q., Hu C., Gu J., Tian Z. (2016). Frequency-agile electromagnetically induced transparency analogue in terahertz metamaterials. Opt. Lett..

[21] Wu D., Liu Y., Yu L., Yu Z., Chen L., Li R., Ma R., Liu C., Zhang J., Ye H. (2017). Plasmonic metamaterial for electromagnetically induced transparency analogue and ultra-high figure of merit sensor. Sci. Rep..

[22] Rana F., George P.A., Strait J.H., Dawlaty J., Shivaraman S., Chandrashekhar M., Spencer M.G. (2009). Carrier recombination and generation rates for intravalley and intervalley phonon scattering in graphene. Phys. Rev. B.

[23] Yee K.-J., Kim J.-H., Jung M.H., Hong B.H., Kong K.-J. (2011). Ultrafast modulation of optical transitions in monolayer and multilayer graphene. Carbon.

[24] Li W., Chen B., Meng C., Fang W., Xiao Y., Li X., Hu Z., Xu Y., Tong L., Wang H. (2014). Ultrafast all-optical graphene modulator. Nano Lett..

[25] Schedin F., Geim A., Morozov S., Hill E., Blake P., Katsnelson M., Novoselov K. (2007). Detection of individual gas molecules adsorbed on graphene. Nat. Mater..

[26] Koppens F.H., Chang D.E., Garcia de Abajo F.J. (2011). Graphene plasmonics: A platform for strong light–matter interactions. Nano Lett..

[27] Ju L., Geng B., Horng J., Girit C., Martin M., Hao Z., Bechtel H.A., Liang X., Zettl A., Shen Y.R. (2011). Graphene plasmonics for tunable terahertz metamaterials. Nat. Nanotechnol..

[28] Vakil A., Engheta N. (2011). Transformation optics using graphene. Science.

[29] Zhao X., Yuan C., Zhu L., Yao J. (2016). Graphene-based tunable terahertz plasmon-induced transparency metamaterial. Nanoscale.

[30] Zhao X., Yuan C., Lv W., Xu S., Yao J. (2015). Plasmon-induced transparency in metamaterial based on graphene and split-ring resonators. IEEE Photonics Technol. Lett..

[31] Ding J., Arigong B., Ren H., Zhou M., Shao J., Lu M., Chai Y., Lin Y., Zhang H. (2014). Tuneable complementary metamaterial structures based on graphene for single and multiple transparency windows. Sci. Rep..

[32] Tang W., Wang L., Chen X., Liu C., Yu A., Lu W. (2016). Dynamic metamaterial based on the graphene split ring high-q fano-resonnator for sensing applications. Nanoscale.

[33] He X., Lin F., Liu F., Shi W. (2016). Terahertz tunable graphene fano resonance. Nanotechnology.

[34] Liu C., Zha S., Liu P., Yang C., Zhou Q. (2018). Electrical manipulation of electromagnetically induced transparency for slow light purpose based on metal-graphene hybrid metamaterial. Appl. Sci..

[35] Liu T., Zhou C., Cheng L., Jiang X., Wang G., Xu C., Xiao S. (2018). Actively tunable slow light in a terahertz hybrid metal-graphene metamaterial. arXiv.

[36] Zhang Z., Yang J., He X., Han Y., Zhang J., Huang J., Chen D., Xu S. (2018). Active control of broadband plasmon-induced transparency in a terahertz hybrid metal–graphene metamaterial. Rsc Adv..

[37] He X., Huang Y., Yang X., Zhu L., Wu F., Jiang J. (2017). Tunable electromagnetically induced transparency based on terahertz graphene metamaterial. Rsc Adv..

[38] Lao C., Liang Y., Wang X., Fan H., Wang F., Meng H., Guo J., Liu H., Wei Z. (2019). Dynamically tunable resonant strength in electromagnetically induced transparency (eit) analogue by hybrid metal-graphene metamaterials. Nanomaterials.

[39] Xiao S., Wang T., Liu T., Yan X., Li Z., Xu C. (2018). Active modulation of electromagnetically induced transparency analogue in terahertz hybrid metal-graphene metamaterials. Carbon.

[40] Yan X., Wang T., Xiao S., Liu T., Hou H., Cheng L., Jiang X. (2017). Dynamically controllable plasmon induced transparency based on hybrid metal-graphene metamaterials. Sci. Rep..

[41] Ordal M.A., Bell R.J., Alexander R.W., Long L.L., Querry M.R. (1985). Optical properties of fourteen metals in the infrared and far infrared: Al, Co, Cu, Au, Fe, Pb, Mo, Ni, Pd, Pt, Ag, Ti, V, and W. Appl. Opt..

[42] He X. (2015). Tunable terahertz graphene metamaterials. Carbon.

[43] Vasić B., Jakovljević M.M., Isić G., Gajić R. (2013). Tunable metamaterials based on split ring resonators and doped graphene. Appl. Phys. Lett..

[44] Huang Y., Yao Z., Hu F., Liu C., Yu L., Jin Y., Xu X. (2017). Tunable circular polarization conversion and asymmetric transmission of planar chiral graphene-metamaterial in terahertz region. Carbon.

[45] Xiao S., Wang T., Liu Y., Xu C., Han X., Yan X. (2016). Tunable light trapping and absorption enhancement with graphene ring arrays. Phys. Chem. Chem. Phys..

[46] Xiao S., Wang T., Jiang X., Yan X., Cheng L., Wang B., Xu C. (2017). Strong interaction between graphene layer and fano resonance in terahertz metamaterials. J. Phys. D Appl. Phys..

[47] Luk’yanchuk B., Zheludev N.I., Maier S.A., Halas N.J., Nordlander P., Giessen H., Chong C.T. (2010). The fano resonance in plasmonic nanostructures and metamaterials. Nat. Mater..

[48] Lu Y., Xu H., Rhee J.Y., Jang W.H., Ham B.S., Lee Y. (2010). Magnetic plasmon resonance: Underlying route to plasmonic electromagnetically induced transparency in metamaterials. Phys. Rev. B.

[49] Fleischhauer M., Imamoglu A., Marangos J.P. (2005). Electromagnetically induced transparency: Optics in coherent media. Rev. Mod. Phys..

[50] Yannopapas V., Paspalakis E., Vitanov N.V. (2009). Electromagnetically induced transparency and slow light in an array of metallic nanoparticles. Phys. Rev. B.

[51] Luo W., Cai W., Xiang Y., Wang L., Ren M., Zhang X., Xu J. (2016). Flexible modulation of plasmon-induced transparency in a strongly coupled graphene grating-sheet system. Opt. Express.

